# Variability in clinicians’ understanding and reported methods of identifying high-risk surgical patients: a qualitative study

**DOI:** 10.1186/s12913-020-05316-0

**Published:** 2020-05-15

**Authors:** Amanda Selwood, Brette Blakely, Siva Senthuran, Paul Lane, John North, Robyn Clay-Williams

**Affiliations:** 1 Centre for Healthcare Resilience and Implementation Science, Australian Institute of Health Innovation, Macquarie University, Level 6, 75 Talavera Road, Macquarie Park, NSW 2109 Australia; 2 Townsville Hospital and Health Service, 100 Angus Smith Drive, Douglas, QLD 4814 Australia; 3 College of Medicine & Dentistry, James Cook University, Townsville, QLD 4811 Australia; 4 Princess Alexandra Hospital, 199 Ipswich Rd, Woolloongabba, QLD 4102 Australia

**Keywords:** Surgery, High-risk patients, Frailty, Qualitative research, Patient risk, Shared decision-making

## Abstract

**Background:**

High-risk patients presenting for surgery require complex decision-making and perioperative management. However, given there is no gold standard for identifying high-risk patients, doing so may be challenging for clinicians in practice. Before a gold standard can be established, the state of current practice must be determined. This study aimed to understand how working clinicians define and identify high-risk surgical patients.

**Methods:**

Clinicians involved in the care of high-risk surgical patients at a public hospital in regional Australia were interviewed as part of an ongoing study evaluating a new shared decision-making process for high-risk patients. The new process, Patient-Centred Advanced Care Planning (PC-ACP) engages patients, families, and clinicians from all relevant specialties in shared decision-making in line with the patient’s goals and values. The semi-structured interviews were conducted before the implementation of the new process and were coded using a modified form of the ‘constant comparative method’ to reveal key themes. Themes concerning patient risk, clinician’s understanding of high risk, and methods for identifying high-risk surgical patients were extricated for close examination.

**Results:**

Thirteen staff involved in high-risk surgery at the hospital at which PC-ACP was to be implemented were interviewed. Analysis revealed six sub-themes within the major theme of factors related to patient risk: (1) increase in high-risk patients, (2) recognising frailty, (3) risk-benefit balance, (4) suitability and readiness for surgery, (5) avoiding negative outcomes, and (6) methods in use for identifying high-risk patients. There was considerable variability in clinicians’ methods of identifying high-risk patients and regarding their definition of high risk. This variability occurred even among clinicians within the same disciplines and specialties.

**Conclusions:**

Although clinicians were confident in their own ability to identify high-risk patients, they acknowledged limitations in recognising frail, high-risk patients and predicting and articulating possible outcomes when consenting these patients. Importantly, little consistency in clinicians’ reported methods for identifying high-risk patients was found. Consensus regarding the definition of high-risk surgical patients is necessary to ensure rigorous decision-making.

## Background

Patients presenting for elective surgery with a higher than usual risk of adverse outcomes require extensive and considered decision-making regarding their care. In recent years, there has been a move towards shared decision-making with these patients to ensure their care aligns with their values and goals [1–6]. For instance, Patient-Centred Advanced Care Planning (PC-ACP) is a shared decision-making process aiming to create multidisciplinary advanced care plans for high-risk surgery patients [7]. This process engages clinicians across disciplines and specialties alongside patients and families in a collaborative framework. PC-ACP explores patients’ goals and values and facilitates discussion on the degree to which surgery aligns with those goals. PC-ACP is currently being implemented in an acute care hospital in regional Australia.

For high-risk patients to be afforded the opportunity to engage in meaningful shared decision-making in processes like PC-ACP, they need to be identified as high-risk before surgery is offered. Identifying high-risk patients early in the decision-making process means alternative treatments can be considered. If surgery is the preferred choice, steps may be taken to reduce the risk of adverse or undesired outcomes while considering the proposed procedure, the reason for elevated patient risk, and a full understanding of patient preferences and needs. In other words, patients must be identified as high-risk early enough in their care to participate in care pathways such as PC-ACP.

However, surgeons’ perceptions, predictions and preferences regarding risk may depend on the individual surgeon [8]. Surgeons vary in their ratings of risk and their decision to perform surgery, even when given the same clinical information [9, 10]. This variability means that a patient may be considered too high-risk to operate on by one surgeon, but of reasonable operative risk by another surgeon, even for the same procedure. Patient risk should not only be assessed by the surgeon: preoperative screening procedures should look at anaesthetic risk also before surgery is scheduled [11]. If the preoperative screening flags a patient as requiring further assessment, an anaesthetist should also assess the patient. Therefore, even after a patient has been offered surgery, an anaesthetist may judge a patient’s anaesthetic risk to be too high for the surgery to take place.

Variability in risk judgements and decisions to operate have led to the development and use of screening tools for frailty or overall patient risk [12–15]. There are multiple screening tools available [12–20]. However, many only apply to specific types of patients, procedures or sources of risk. In addition, clinicians may not have the resources or make the time to apply the screening tool accurately, particularly for the most comprehensive assessments [21]. Therefore, formalised screening tools may not be commonly used [6].

Here we report how clinicians at a regional Australian hospital involved in treating high-risk surgical patients define and identify patients as high-risk in their everyday practice. This acute care hospital is the only tertiary facility in a large area of rural Queensland that treats frail and high-risk surgical patients. The hospital is currently engaged in using PC-ACP to improve the decision-making process for these patients [7]. The interviews were conducted prior to the implementation of PC-ACP.

## Methods

### Study setting, participant recruitment and data collection

The study was conducted at a tertiary centre in North Queensland, Australia. The hospital receives patients from an area of 750,000 km^2^, with many remote patients receiving telehealth services or being transferred from smaller, rural hospitals.

Ethical approval for this study was obtained from the Townsville Hospital and Health Service Human Research Ethics Committee [HREC/16/QTHS/100]. Participants were clinicians working in the Department of Surgery at Townsville Hospital. They were purposively selected by two of the researchers (SS, RCW) to represent all relevant professions involved in treating high-risk surgical patients, including surgeons, anaesthetists, intensivists, and nursing and administrative staff, and invited face-to-face to participate in the study. Those who elected to participate were invited to complete a survey and to participate in a semi-structured interview at their workplace. Data collection was conducted per the method outlined in the published study protocol, [7] prior to implementation of a Patient-Centred Advanced Care Planning (PC-ACP) intervention.

Two postdoctoral research academics, who were experienced in qualitative methodologies, conducted the interviews (AS, RCW). No relationship was established between the interviewers and participants prior to conducting the interviews. An interview schedule was developed specifically for the purposes of this study. Participants were asked about their experience and views on shared decision-making with high-risk surgical patients (see Additional File 1 for Interview guide). Additional questions were asked as needed for clarification, or to follow up on important points. Additional participants were recruited and interviewed until all professions were represented, or theoretical saturation was reached.

### Data management and analysis

Interviews were audio recorded and professionally transcribed verbatim. Analysis was conducted on interview transcripts only; no field notes were taken during or after the interviews. Transcripts were not returned to participants for comment or correction. Transcripts were subjected to inductive interpretive analysis in NVivo 11 (QSR International, Melbourne, Australia), using a modified form of the ‘constant comparative method’ to identify key themes by one of the research academics who had conducted the interviews [22, 23]. According to this method, the data were organised and used to explore connections between data elements and to develop sets of concepts. Once coded, segments of data were formally linked, allowing themes to emerge.

## Results

Thirteen clinicians and administrative staff were interviewed in single sessions, face-to-face over 5 days in November 2016. Average interview length was 27 min (range 16–43 min). All invited participants consented to be interviewed, and none subsequently withdrew from the study. Participant demographics are presented in Table 1. The sample represented all the specialties treating high-risk surgical patients (e.g. general, cardiac, orthopaedic surgery, etc.), although some specialties were only represented by one or two clinicians. As the study is ongoing participants were not asked to provide feedback on the findings presented here.

**Table 1:** Participant demographics

	Number of participants
Total N (M: F)	13 (10:3)
Age	
35 to 44	7 (54%)
45 to 54	2 (15%)
55 to 64	3 (23%)
Not specified	1 (8%)
Profession	
Surgeon	5 (38%)
Anaesthetist	2 (15%)
Intensivist	2 (15%)
Anaesthetist/Intensivist	2 (15%)
Nursing and administrative staff	2 (15%)
Professional level	
Consultant	8 (62%)
Senior Medical Officer	3 (23%)
Registered Nurse	2 (15%)
Average time since qualifying	12.5 years (1–29)
Average time at current employer	9.6 years (1.5–28)

### Analysis

Table 2 shows the four themes that emerged from the coded interviews. Of immediate note was the importance and variance within the theme: factors involved in patient risk — which warranted further exploration and is the focus of this paper. On further analysis, six sub-themes emerged within this theme. The six sub-themes are presented in Table 3 and described below. Analysis of the remaining three of themes (shared decision-making, stakeholder relationships and anticipated PC-ACP implementation) will be presented separately, with analysis of post-implementation interviews.

**Table 2:** Coding themes from participant interviews

Theme	Description
Shared decision-making	Perceptions and experience of shared decision-making with other clinicians, patients and their families.
Stakeholder relationships	Communication with, attitudes towards and relationships with other clinicians, patients and family members that was no specifically related to shared decision-making.
Factors involved in patient risk	Defining, identifying and caring for high-risk surgical patients.
Anticipated PC-ACP implementation	Views on PC-ACP prior to implementation, based on viewing model of the new process, including expectations on how it will work and requirements for its success.

**Table 3:** Coding sub-themes within Factors in Patient Risk

Sub-theme	Description
Increase in high-risk patients	Increasing numbers of high-risk patients presenting for or being offered surgery and the reasons for and implications of this change
Recognising frailty	Difficulties in defining and recognising frailty in surgical patients
Risk-benefit balance	Balancing the risks of surgical intervention with its potential benefits and the risks of no surgical intervention
Suitability and readiness for surgery	The question of whether a patient is suitable for surgery or the procedure, now or at a later date
Avoiding negative outcomes	Avoiding adverse outcomes of surgery as well as complaints and legal action resulting from adverse outcomes
Methods in use for identifying high-risk patients	How participants report identifying high-risk patients in their clinical practice

### Recognising frailty

Participants disagreed on clinicians’ ability to recognise frailty. One participant stated that anaesthetists are the most proficient at recognising frailty, even if other specialties use the same criteria (Table 4, quotes 6 to 7). Other participants believed that frailty is both poorly understood and recognised in medicine. Hence, frailty is often underestimated in practice (Table 4, quotes 8 to 9). This view was corroborated by the fact that patients from remote areas or communities are often not recognised as frail until they arrive at the hospital for their surgery (Table 4, quote 10). Therefore, clinicians may base their frailty assessments on perceptual cues (see ‘Methods in use for identifying high-risk patients’ below).

**Table 4:** Participant quotes on risk

Theme	Quote
Increase in high-risk patients	1. … if you’ve got a patient who has got significant comorbidities, the sorts of patients we’re increasingly seeing, if you’ve got patients where the surgery is going to be particularly major, say oesophagostomy, cardiac surgery, pancreatic Whipple’s procedure, then certainly the outcomes for the patients are very, very different. [Intensivist 4]
	2. I think this is one thing which has been discussed for quite some time, one, because of the ageing population and also a lot more obese people we deal with. [Surgeon 2]
	3. … dilemmas of patients nowadays living well past their 80s, 90s, and we even have hundred-year-olds, so when do you stop? Do you just go on their chronological age and stop, or a patient needs - got an aneurysm, needs a complex procedure and the patient is 95, should we let them die just because they’re 95? Or should we do a complex operation to save them? [Surgeon 2]
	4. Not common, but I think we’re seeing a lot more high-risk patients now because we’re all getting older and people are living longer, so we are seeing a lot more patients that are high-risk that we wouldn’t have operated on in the past, due to great medical advances and all of that. [Nurse/admin 1]
	5. Back in the old days the minute you hit 80 you got this [risk assessment], whether you had no other things, but we don’t necessarily do that now because some of the 80 years olds walk in better than the 60-year olds. [Nurse/admin 1]
Recognising frailty	6. I think more and more people are realising and more and more surgeons are realising that anaesthetics is not just getting someone off to sleep and waking them up at the end of the day. It’s - it kind of seeps into the fields of perioperative medicine, which involves identifying which patients are frail and - because, yeah, some surgeons are good at identifying those. Some may not be. [Anaesthetist 2]
	7. I think frailty is frailty. At the end of the day, it’s the patient. If a patient is frail, he’s not only frail for the surgeons in one aspect and then a different aspect for - as an anaesthetist or an intensivist. The frailty is going to be because of the same reasons. [Anaesthetist 2]
	8. I think it says at the top there it’s about recognising frailty and it’s something that we as a profession, I’m talking medicine, not particular subgroups, have done particularly poorly. [Intensivist 4]
	9. So, I guess people might look at level of function or just a list of comorbidities generally. I think frailty is often poorly recognised and poorly understood by surgeons and to some extent anaesthetists as well. [Intensivist 2]
	10. Yes, and I mean often we can hear a story from [remote cities and towns], whether this patient’s this, this, this; then they turn up on your doorstep and they’re on their wheelie walker or they - and so the story completely changes. They might not have been high-risk before and then you’ve eyeballed them and go, no. [Nurse/admin 1]
Risk-benefit balance	11. … it’s the small, high-risk patients, especially if you have a procedure which might be of marginal overall benefit to the patient. It doesn’t mean that they can’t get benefit from it, and it doesn’t mean that we should necessarily deny them their opportunity if they are genuinely miserable with their current situation. [Surgeon 1]
	12. I calculated the EuroSCORE, and it came to about 35% 40% mortality. ... So, I went back to the surgeon. I said, do you think you really should be operating on this patient with such a high mortality rate? He just looked at me and said, if I don’t operate this patient, her mortality is 100%. [Anaesthetist 2]
	13. What’s best for the patient may not necessarily be the most that we can do. In some areas in medicine doing nothing may be the best thing. ... Think simple, aiming for comfort, palliative approach may be the best thing. [Intensivist 1]
	14. I guess it comes back to the constant of futility. Yes, we can do operations, and we can do all these other things, but is it really going to benefit the patient? [Intensivist 4]
Suitability and readiness for surgery	15. I have been involved in one or instances where it was extremely clear cut that that patient would not even be fit for a haircut - let alone even a palliative non-curative surgery. [Anaesthetist 2]
	16. So, when we make decisions about whether a patient is appropriate for this procedure or that procedure, when we make a decision about whether a patient should have an operation at all, when we make decisions about whether the patients should go to intensive care, should they go to intensive care for a short period of time. So when we make a decision with their treating physicians about whether now is the right time for surgery or whether that should be done in the future. [Anaesthetist 1]
	17. They look at us as the primary and the initial gate, if you were to call it, as to see whether this patient is really - has that reserve to actually undergo this procedure. When I say reserve, it’s for us - predominantly, it’s physiological reserve, but I - personally I look at the patients as a whole, so it’s also the psychological, the social aspects of whether they can actually - anybody can operate and we can give them an answer, they can - yes, they’ll get better, but are they actually able to go back home? Is there enough support for them? I think in terms of looking at the whole package. [Anaesthetist 2]
	18. Because some people are happy with their quality of their life and they might decide at this stage I’m not ready, I don’t want to go ahead but then come back in 3 months and go, alright, I have thought about it, and now I’m ready. So, I think you’ve got to wait till they’re mentally ready. [Nurse/admin 1]
Avoiding negative outcomes	19. Hopefully, we’ll avoid unnecessary surgery, we’ll avoid unwise surgery, and we’ll avoid bad outcomes that can be avoided. So, we’ll have more advanced and sensible discussions about likely outcomes, so people will have a more realistic, potentially, expectation of what their outcomes are likely to be and make more realistic decisions about those things. [Anaesthetist 1]
	20. A lot of patients think they’ll either survive the surgery and be okay or not survive the surgery and then it won’t matter. [Intensivist 3]
	21. It’s pretty, very uncommon for a patient to die on the table. But someone who doesn’t have the reserves to recover from an operation, particularly if there’s complications, it’s going to be in the post-operative period that we’re struggling, and it may well be a patient’s being supported in intensive care but what treatment’s appropriate and what are the goals of treatment. If we’ve had those discussions before the operation, it’s useful. So that is something I do sometimes. [Surgeon 3]
	22. … we want patients to be satisfied with what we do, and even if they don’t get an optimal result or the end of it, complication, they can at least say that, okay well we went through this process and I’m just unlucky [Surgeon 5]
Methods in use for identifying high-risk patients	23. Well, the surgeon normally flags that that they’re high-risk, and then from there we’ll get the anaesthetist and the intensivist involved. [Nurse/admin 1]
	24. Well, we have some vague indicators. I think it’s just experience mainly. But then we have some indicators like for cardiac surgery there is an indicator for EuroSCORE. So, we put all the patient details, and that gives us a mortality. So, if the mortality is more than say 15%, then we know that the patient is high-risk. [Surgeon 4]
	25. So, there are some validated tools, which are available. I’ve never had to use any of them. [Anaesthetist 2]
	26. Well, you sort of - a lot of it will come from their history. So, if they have severe cardiac or similar problems, they’re morbidly obese and poorly mobile, so if they come in in a wheelchair because they can’t really mobilise. You see people who still manage to mobilise with fairly severely arthritic joints, for example, but it just means that - or it suggests that their reserves are not so good, if they turn up like that. So, they would probably be the ones, so someone with cardiac problems, obese and turns up in a wheelchair. [Surgeon 1]
	27. Oh, we pretty know who the high-risk patient. We know from the - well it will be several things. It’s usually patient-related factors like old age, frailty, number of cardiac problems like patient needs bypass, multiple valves need to be done, patient’s heart function isn’t particularly good. Then you look at other organ functions, lungs, patients who have got emphysema or other lung disease for that matter. Kidney’s, patients on dialysis, they are always high-risk. [Surgeon 5]
	28. They’re not always old. We get some really frail people in their forties, so they had rheumatic fever and other things, they look physically older than what they are. So chronologically, they don’t have to be that old. They can be quite young but have been, I guess, disadvantaged when the genes were handed out. [Intensivist 4]
	29. But it’s really not an art, it’s just experience and pattern recognition and putting this - trying to see the most similar situation that you’ve been in before that might offer the patient the best outcome. [Intensivist 3]
	30. You just know it. You just know. You look at - we call it an end-of-bed-o-gram. Right? So, you stand at the end of the bed, and you just get a brief idea - just looking at everything - and of course, you do need to delve into a few more specifics and details later, but you get that idea about who’s likely to make it and who’s going to struggle. [Anaesthetist 2]
	31. There’s something called eyeball test in med surgery. I don’t know if you heard or not. You look at the patient from end of the bed, and it doesn’t look like 80 or 85-year-old woman. Little old woman, frail looking. [Surgeon 5]
	32. Can I quote The Castle? ... It’s the vibe. [Intensivist 2]
	33. I take all the patients I’m planning to do a big operation on, I walk them up three flights of stairs. So as part of coming to see me in clinic either myself or if I think - I do it personally myself if I think they really are pretty borderline, if they look fairly fit, I’ll often get the resident to do it. Basically, they need to be able to walk up three storeys and chat to me at the top. [Surgeon 3]

### Risk-benefit balance

Study participants perceived that operative risk was a directly related to the individual patient and the proposed procedure. To them, decision-making rested on a balance between perceived risk and potential benefit of the procedure for the patient (Table 4, quote 11). Participants noted this balance to be unique to each case. Determinants included patient expectations and goals, the patient’s condition, the procedure and the expected outcomes of the surgery. In circumstances where the surgery was likely to be curative, or when the disease process was more dangerous than the procedure, participants favoured surgery (Table 4, quote 12). However, if the surgery was considered to be marginally beneficial, participants generally favoured a palliative approach (Table 4, quotes 13 to 14).

### Suitability and readiness for surgery

Clinicians cited various considerations in patients’ suitability for surgery. The first was after careful consideration of the diagnosis, whether the patient is fit for surgery at all (Table 4, quotes 15 to 16). The second was whether the proposed procedure or a less invasive procedure is preferable (Table 4, quote 16). The third was the optimal timing of the surgery for the patient (Table 4, quote 16). Factoring into these considerations were the patient’s likelihood of surviving the operation, the postoperative possibilities and their ability to recover from it (Table 4, quote 17). Patients’ ability to recover from surgery depended on both their physical and psychological health (Table 4, quotes 17 and 18). Social factors including living conditions and social support were also raised as contributing to patients’ suitability for surgery (Table 4, quote 17).

### Avoiding negative outcomes

Identifying high-risk patients is essential to minimise the likelihood of adverse or undesirable outcomes (Table 4, quote 19). Most patients only consider one adverse outcome – that of not surviving the surgery (Table 4, quote 20). In contrast, clinicians cited gradual postoperative deterioration or experiencing poorer health than before surgery as more common adverse outcomes. Thus, surgeons reported asking ICU colleagues to assess patients’ ability to recover from surgery and possible postoperative needs (Table 4, quote 21). Another negative outcome reported was patient complaints or legal action. Participants believed that informing patients comprehensively about their high risk before surgery would mitigate against complaints and legal action if adverse outcomes occurred (Table 4, quote 22).

### Methods in use for identifying high-risk patients

Participants agreed that surgeons would be the profession most likely to identify a high-risk patient in the first instance (Table 4, quote 23). However, participants believed that high-risk patients ‘missed’ by the surgeon would be ‘caught’ by anaesthetists or intensivists.

Three main methods of identifying high-risk patients were reported: patient pathology, screening tools, patient characteristics, and other informal methods. Nine of 13 participants named specific screening tools to identify high-risk patients. However, there was no consensus on which screening tools should be used, and not all who suggested a screening tool reported using it (Table 4, quotes 24 to 25). The most common screening tool, suggested by four clinicians, was the EuroSCORE (European System for Cardiac Operative Risk Evaluation) for cardiac patients [13, 19]. The second most common was the clinical frailty scale developed by the Canadian Study on Health & Aging, suggested by two clinicians [14]. Other formal tools included American Society of Anaesthesiologists (ASA) grades, the Society of Thoracic Surgeons (STS) online risk calculator, and an integer risk score developed by researchers from the University of Florida (UF score) [15, 18, 20]. Non-validated tools mentioned included standard hospital health screening questionnaires, angiograms, blood tests, and medical indicators of end-stage renal failure.

Individual patient characteristics were the most commonly suggested means of identifying high-risk patients. These can be further categorised into eight characteristics: number of comorbidities, type of comorbidity, patient history, frailty, poor mobility, age, obesity and presence of terminal illness (Table 4, quotes 26 to 28). Table 5 shows the number of participants who reported using each characteristic alongside illustrative examples.

**Table 5:** Patient characteristics associated with high risk reported by participants

Patient characteristic indicating frailty	Number of participants reporting (*N* = 13)	Examples
Number of comorbidities	7	Multi-system disease, 2–3 comorbidities, number of cardiac problems
Type of comorbidity	6	COPD, cardiac problems, hypertension, diabetes
Patient history	4	Previous heart attacks, multiple surgeries
Frailty	4	Frail, weight loss, fatigue, level of function, frequent falls, frequent infections
Poor mobility	3	Use of walking frame, use of wheelchair
Age	3^a^	Ageing population, elderly
Obesity	2	Obese patients, morbidly obese
Terminal illness	1	Terminal multi-organ failure

^a^An additional 3 participants stated that age was not necessarily an indicator of risk

Four of the participants reported making a ‘global’ assessment of the patient. This method of identifying high-risk patients appeared to rely on a subjective judgement of frailty (Table 4, quotes 29 to 32). Another surgeon screened out patients unfit for high-risk surgeries by asking them to walk up three flights of stairs and be able to speak at the top (Table 4, quote 33). Thus, methods for identifying high-risk patients were varied, sometimes ad hoc, and frequently based on clinicians’ previous experiences and personal preferences.

## Discussion

The balance between the risks and benefits of surgery is vital for decision-making for high-risk patients [6]. As participants noted, surgery is only appropriate if the benefits of surgery outweigh the potential risks. However, studies on surgeons’ decision-making suggest that surgeons differ in their assessment of risk and their likelihood of recommending surgery given the same objective risk [9, 24].

Although the clinicians interviewed in our study all worked in the same hospital department, the methods they used to identify high-risk patients were not consistent. It is true that participants included surgeons from different specialties (including cardiac, general and orthopaedic surgery), who may treat patients with different kinds of risk and perform procedures with varying levels of risk. However, the anaesthetists and intensivists who were interviewed treated patients receiving all kinds of surgery, and there was as much variability in their methods of identifying high-risk patients as in surgeons’ methods. Moreover, surgeons within the same specialty were not necessarily consistent in their methods either, even though given our small sample, some specialties were only represented by one or two surgeons.

Participants often used their own judgement to identify high-risk patients, particularly when identifying patients who are high-risk due to frailty. These judgements were based on their personal experience, heuristics and preferences. Identifying frailty based on personal experience and overall presentation of a patient appears to be common [6, 8, 16]. These subjective methods rely on visual observation, which may lead to frailty being under-recognised. If clinicians have a variable understanding of frailty and recognise frailty in different ways, frailty may be ‘missed’ if it does not present in a manner that aligns with the experience of the assessing clinician. Therefore, subjective assessments of frailty are likely to be inadequate if frailty is as poorly understood or under-recognised as participants claimed. Remote patients may be especially disadvantaged by these methods because they are not likely to be identified as frail by clinicians using visual assay methods until they arrive at the hospital. Therefore, their surgical treatment may be revised, delayed or cancelled at the last minute. This difficulty may not necessarily be solved by using objective measures, as many of these need to be administered in person by a qualified physician [12]. These issues must be considered in implementing systematic shared decision-making processes specifically for high-risk patients such as PC-ACP.

Clinicians’ inadequate understanding and under-recognition of frailty is supported by the literature. Medicine appears to lack a clear definition of frailty [21, 25]. For instance, while clinicians generally agree that frailty is indicated by the presence of low physiological reserve, published definitions do not always include deterioration and weakness [26–28]. This ambiguity is complicated by the existence of several types of frailty, each focusing on different criteria. For example, phenotype definitions of frailty (weight loss, exhaustion, slow gait, weak grip strength and low activity) take into account different criteria to functional or ‘multidomain’ definitions; although they are moderately related, they are different constructs [26, 29, 30]. Some frailty measures, such as the Frailty Index from the Canadian Study of Health and Aging take into account factors such as comorbidity, whereas others, such as the Edmonton Frail Scale do not [12, 14]. Finally, another contributing factor is the heterogeneity among frail patients [31, 32]. Even published studies on frailty often identify frailty without objective measures [16]. Given such a diversity in the definition, measurement, and presentation of frailty, it is not surprising that it may be under-recognised.

Using objective risk scales and frailty assessment techniques, such as the Edmonton Frail Scale or those described by participants, is clearly preferable to subjective measures [6, 13–20]. However, as demonstrated above, objective frailty measures vary in their criteria, so finding the most accurate or useful measure may be difficult, and some risk assessment techniques may be more suitable for particular kinds of procedures [16]. Therefore, the questions of which assessment tool should be used, which patients should be assessed, and how assessments should be administrated to patients from remote areas or communities, need to be addressed before hospitals mandate that surgeons use objective scales to identify high-risk patients. The cultural barrier, whereby participants appeared to trust their own judgement of a patients’ frailty more than an objective assessment on paper, also needs to be overcome.

Patients with risk factors that are available to the surgeon at consultation, such as comorbidities, advanced age, obesity and complicated medical history, appear to be comparatively easy to identify as being high-risk. This ease appears to be due to hospital pre-screening processes, which specifically ask about these risk factors, as well as the fact that these risk factors are relatively well-defined and unambiguous. For instance, comorbidities such as atrial fibrillation stroke risk are already assessed using objective scales such as CHADS_2_ and CHA_2_DS_2_-VASc for [33, 34]. However, without adequate communication with a patient and their family, risk factors for comorbidities can still be missed, leading to negative outcomes [35]. Moreover, the disagreement among participants over age being a reliable indicator of patient risk suggests that identifying high-risk patients using these factors as criteria is still not straightforward. The argument over the relationship between age and frailty is reflected in literature findings: on the one hand, being over 80 years of age is a predictor of perioperative complications and length of stay [36]. On the other hand, frailty measured by the Frailty Index from the Canadian Study of Health and Aging is a better predictor of hospital complications than age [12, 28]. Furthermore, mortality rates for patients aged over 80 are extremely low for some procedures [37]. If this is the case, age may not be as important a risk factor as frailty.

Further complicating the issue of frailty is the recognition that medical frailty, even if clearly defined, is not the only important factor in assessing patient risk. Participants attested that patients’ suitability and readiness for surgery is based on not only on physiological, but also psychological and social factors. Thus, a surgeon can consider a patient as suitable (or not suitable) for surgery based on their assessment of risk and benefit, and also ready (or not ready) for surgery based on the information the patient has given them about their psychosocial situation. However, one participant noted that patients are more likely to divulge relevant psychosocial information to nursing staff than to their surgeon. If this is the case, this means it is important to involve nursing staff in discussions about a high-risk patients’ suitability for surgery. Such factors have implications for any attempts to implement frailty screening measures.

Each of the above factors is important to avoid negative outcomes for patients. As participants suggested, ensuring patients have a realistic understanding of expected outcomes and potential risks before their procedure may increase their satisfaction, [38] reducing complaints and litigation. High-risk patients need a thorough understanding of their high-risk status, the procedure and potential outcomes in order to have informed consent and fully engage in shared decision-making about their treatment [39–41]. For certain surgical procedures, it may even be advisable to include an assessment of patient risk along with any objective risk scores or frailty assessments in the consent form, to ensure high-risk patients are identified prior to consent.

**Fig. 1:**
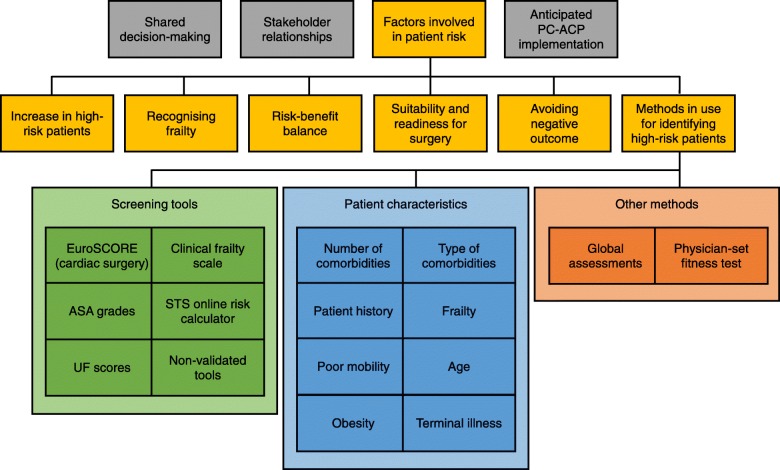
Synthesis of sub-themes within factors related to patient risk

Figure 1 synthesises the sub-themes within factors related to patient risk as well as how they relate to each other and to the central factor, high-risk surgical patients. The increase in high-risk patients means that factors involved in patient risk are becoming more central to surgical decision-making, especially the ‘decision to operate’. A critical component of identifying high-risk patients is successful recognition of frailty. High-risk patients need to be recognised in order to consider their suitability and readiness for surgery, and the risk-benefit balance of the proposed procedure. It is crucial to give these considerations enough weight in order to avoid negative outcomes. These can be more complex than not surviving the surgery.

As participants noted, high-risk patients are presenting for surgery in increasingly larger numbers. The literature supports this observation. In line with participant accounts, the literature often cites the ageing population as the primary reason for increasing numbers of high-risk surgical patients [21, 28, 29, 42, 43]. The literature also supports the increase in comorbidities being a factor in the upsurge of high-risk surgical patients [3, 44–46]. The increase in numbers of patients with significant obesity has resulted in more high-risk patients presenting for surgery, not just for bariatric procedures, but for all types of surgery [47]. Advances in the field of surgery, in particular minimally invasive surgical procedures, [48, 49] mean that surgery can be considered for patients who may have been refused surgery in the past. However, this does not entirely compensate for the higher risk in patients with certain types of pathology and may require many procedures, including those which cannot use minimally invasive techniques pose an increasing problem in the future. Therefore, the need to accurately identify frailty early will only become more crucial in the coming years.

### Limitations

This study was conducted as part of an evaluation of PC-ACP, a new decision-making process for high-risk surgical patients currently being implemented in one hospital. Therefore, the study was limited in scope to participants from that hospital. With thirteen participants from a single hospital, this study may not necessarily reflect common practice. This study needs to be replicated on staff involved in high-risk surgery in hospitals across Australia, New Zealand, and other countries to ensure these findings reflect practice more broadly. A larger sample of each profession involved in high-risk surgical patient care, or the inclusion of other professions such as General Practitioners or Care of the Elderly consultants would also be highly valuable in determining the validity of our findings, including whether each of the themes we identified hold true across professions. However, even this small study can contribute to the broader literature on clinicians’ understanding of and methods for identifying high-risk surgical patients.

This study focussed on elective surgery only. However, frailty also needs to be taken into account in emergency surgery. In emergency surgery, the timeframe for decision-making is much shorter than in elective surgery. The shorter timeframe makes it difficult to engage in elaborate decision-making processes or perform lengthy consultations with patients. Thus, identifying high-risk or frail patients in an efficient and accurate way, such as by using objective risk scales or frailty assessments, is crucial. These are already in use by some specialties to quickly identify high-risk patients, such as the P-POSSUM for emergency laparotomy [50, 51].

## Conclusions

This study demonstrated the variability in how clinicians and administrative staff define high surgical risk and identify high-risk patients as they present for surgery. In processes such as PC-ACP, which involve systematic shared decision-making with high-risk surgical patients, clinicians from all specialties, nurses and administrative staff need to have a shared understanding of what a high-risk patient is. In order to drive change in how high-risk surgical patients are identified, agreement needs to be made on not only the definition of frailty, but how it and other risk factors should be assessed. Therefore,. Only then can we be confident that these patients will be adequately informed and involved in the decision-making surrounding their care.

## Supplementary information

**Additional file 1: Appendix 1**. Clinician semi-structured interview questions. This file contains the interview schedule used for the interviews with clinicians and administrators prior to the implementation of PC-ACP.

## Data Availability

The interview transcripts analysed during the current study are available from the corresponding author on reasonable request. The interview questions are available as Additional File 1.

## Abbreviations

ASA: American Society of Anaesthesiologists
EuroSCORE: European System for Cardiac Operative Risk Evaluation
ICU: Intensive Care Unit
PC-ACP: Patient-Centred Advanced Care Planning
STS: Society of Thoracic Surgeons
UF: University of Florida
