# Supplementary material for: Variability in clinicians’ understanding and reported methods of identifying high-risk surgical patients: a qualitative study
Source: BMC Health Serv Res. 2020 May 15;20:427. doi: 10.1186/s12913-020-05316-0 (PMC7227052; doi:10.1186/s12913-020-05316-0)
Supplement: Supplementary file 1 — Additional file 1: Appendix 1. Clinician semi-structured interview questions. This file contains the interview schedule used for the interviews with clinicians and administrators prior to the implementation of PC-ACP. [file 12913_2020_5316_MOESM1_ESM.doc]

**Additional File 1: Appendix 1. Clinician semi-structured interview questions**

Interview Schedule

The information collected will be demographic information and responses to the following questions.

A: Background

- What do you understand by the term ‘shared decision making’?
- Have you had previous experience with shared decision making in the past:
  - Sharing decision making with other clinicians?

Examples, please.

- - Sharing decision making with patients?

Examples, please.

B: Shared decision making

- Do you think it is important to share decision making about patient care with other clinicians? Why/why not?
- Do you think it is important to share decision making about patient care with patients and their families? Why/why not?

C: PC-ACP Process (show clinician diagram and explain the process)

- What are your views on the proposed PC-ACP process?
- Are there advantages to using this process? If so, what are they?
- Are there disadvantages to using this process? If so, what are they?
- Do you see benefits for the patient in using this process? If so, what are they?
- Have you used/tried to use a process similar to this in the past? If so, what were your experiences?

D: Barriers and Enablers

- Did you experience (or anticipate) any barriers to applying the ideas from the PC-ACP process at Townsville Hospital?
- What made it (or would make it easier) to apply the ideas from the PC-ACP process at Townsville Hospital?
- Did you learn (or anticipate learning) anything new, interesting, useful or unexpected as a result of introduction of the PC-ACP process at Townsville Hospital?

E: Sustainability

- Do you believe that the PC-ACP process at Townsville Hospital is sustainable? Why/why not?
- Do you believe that training is required to implement the PC-ACP process at Townsville Hospital? If so, what sort of training and who should be trained?
- Was there any impact of implementation of the PC-ACP process at Townsville Hospital on your job satisfaction?
- Would you like to make any other comments about your experiences in relation to the PC-ACP process at Townsville Hospital?

F: Demographic Information

This information will not be linked to any participant’s name. A number will be assigned as a code reference for analysis purposes.

1.Gender, Age (bracket)

2.Profession (Surgeon, Anaesthetist, Intensivist, Palliative Care, Other)

3.Your professional level at TTHS

4.Time since specialist qualification to practice in this field

5.Time in this organisation
